# Complement C4 Gene Copy Number Variation Genotyping by High Resolution Melting PCR

**DOI:** 10.3390/ijms21176309

**Published:** 2020-08-31

**Authors:** Claudia P. Jaimes-Bernal, Monte Trujillo, Francisco José Márquez, Antonio Caruz

**Affiliations:** 1 Immunogenetics Unit, Department of Experimental Biology, Universidad de Jaén, 23071 Jaén, Spain; cpjaimes@uniboyaca.edu.co (C.P.J.-B.); jmarquez@ujaen.es (F.J.M.); 2 Faculty of Health Sciences, Universidad de Boyacá, 150003 Tunja, Colombia; 3 Transfusion Blood Center, 23009 Jaen, Spain; mariam.trujillo.sspa@juntadeandalucia.es

**Keywords:** copy number variation (CNV), complement C4, DNA copy number variations, real-time polymerase chain reaction

## Abstract

Background: Complement C4 gene copy number variation plays an important role as a determinant of genetic susceptibility to common diseases, such as systemic lupus erythematosus, schizophrenia, rheumatoid arthritis, and infectious diseases. This study aimed to develop an assay for the quantification of copy number variations in the C4 locus. Methods: the assay was based on a gene ratio analysis copy enumeration (GRACE) PCR combined with high resolution melting (HRM) PCR. The test was optimized using samples of a known genotype and validated with 72 DNA samples from healthy blood donors. Results: to validate the assay, standard curves were generated by plotting the C4/RP1 ratio values against copy number variation (CNV) for each gene, using genomic DNA with known C4 CNV. The range of copy numbers in control individuals was comparable to distributions observed in previous studies of European descent. Conclusions: the method herein described significantly simplifies C4 CNV diagnosis to validate the assay.

## 1. Introduction

The complement system is a key element of innate and acquired immunity, and acts as the first line of host defense. *C4* is an essential component of the complement system, acting as an effector protein in the activation of the classical and lectin pathways as a subunit of the C3 and C5 convertases [1]. 

The *C4* gene is located on the long arm of human chromosome 6 (6q21.3) in the major histocompatibility complex (MHC) class III region. This gene has two paralogs *C4A* and *C4B*, sharing 99% sequence identity, each of which is polymorphic in itself. These paralogs have different functional activities and affinities to antigenic surfaces. C4A forms a covalent amide bond with antigens containing amino groups. On the other hand, C4B forms covalent ester bond with targets containing hydroxyl groups. C4A participates also in the withdrawal of immune-complexes through CR1 binding. On the contrary, C4B functions as the main cofactor in the C3 and C5 convertases and the formation of the membrane attack complex [2,3,4,5].

**Figure 1:**
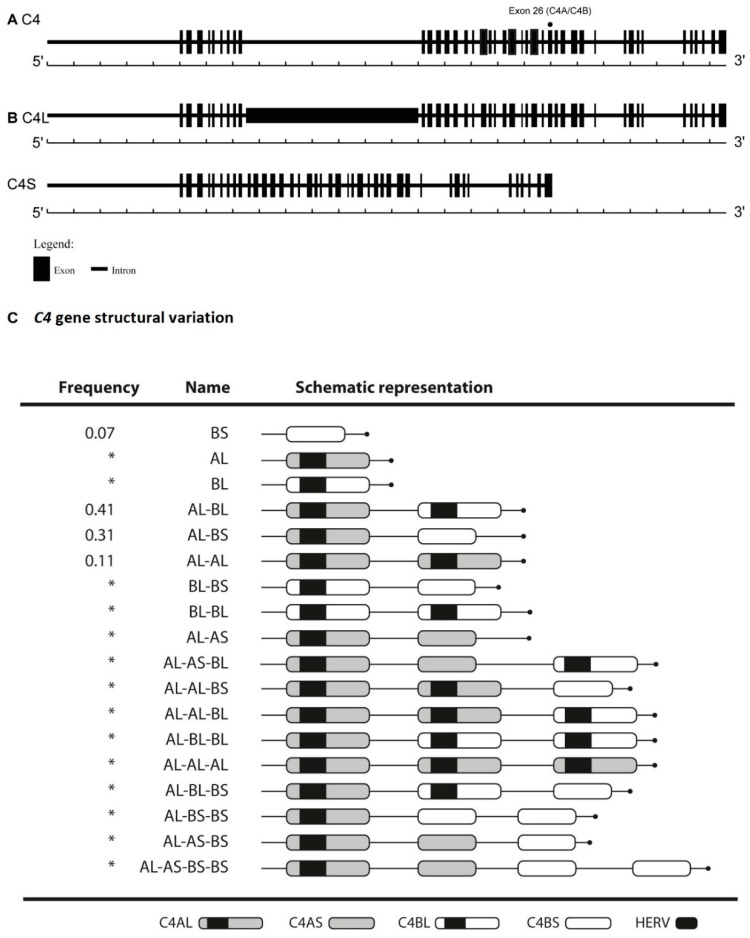
*C4* gene structural variation. (**A**) *C4A* and *C4B* isoforms differ in four amino acids at positions 1101–1106 due to five nucleotide polymorphisms (exon 26). (**B**) The presence or absence of human endogenous retrovirus—6.34kb (HERV) in intron 9, results in *C4L* or *C4S* long *C4* or short *C4* gene, respectively. (**C**) *C4* gene structural variation. AS haplotype carrying only the *C4A* short isoform; BS haplotype carrying only the C4B short isoform; AL haplotype carrying only the *C4A* long isoform; BL haplotype carrying only the *C4B* long isoform. In the figure, all different haplotypes are presented. Marked with an asterisk (*) are the least common in a European-ancestry population.

The *Complement C4* locus exhibits an unusual, complex pattern of genetic diversity, where nonsynonymous SNPs affect protein structure, and insertion-deletion polymorphisms cause frameshifts and premature stop codons. Additionally, *C4* genes harbor polymorphic endogenous retrovirus insertions (HERV-K) that influence mRNA transcription efficiency. Lastly, a complex array of CNV in both *C4A* and *C4B* genes in the region as a result of deletions (null alleles), duplications, and triplications, among others, gives rise to at least 22 haplotypes that include a complex range of genomic structures [6] (Figure 1). 

The total number of *C4* copies varies from two to eight in a diploid genome, corresponding to the sum of *C4A* and *C4B* genes [7]. *C4* diverges also in terms of gene size, 76% of *C4* genes harbor a retrovirus insert, HERV-K, integrated in the ninth intron, giving rise to the long *C4* gene (*C4L*). The remaining 24% of the *C4* genes do not have this retrovirus and are known as short *C4* genes (*C4S*). A wide variation in these polymorphisms according to human populations has been described [8]. Approximately 80% of individuals of European ancestry have three to four *C4* genes. The number of *C4A* gene copies varies between zero to five; for *C4B* between zero to four and for *C4S* and *C4L* genes, respectively, between zero to five copies [7,9] (Figure 1).

Finally, according to their clinical significance, genetic variations have been classified into three categories: benign, pathogenic, and variants of uncertain significance [8]. Moreover, overall *C4*, with its isoforms *C4A*, *C4B* or its gene size variants (*C4S* or *C4L*), gene copy numbers have been associated with disease susceptibilities or differential immune responses. As a case in point, *C4* genes with low copy number are associated with increased predisposition to systematic lupus erythematosus (SLE), rheumatoid arthritis (RA), Grave’s disease, juvenile idiopathic arthritis [10], Schizophrenia [6] or Alzheimer’s disease [11]. Additionally, *C4A* deficiencies are correlated with immunization failure after Hepatitis B vaccination, acute otitis media, sinusitis and pneumonia [12,13,14]. 

Considering the above, different techniques have been used for CNV detection, such as RFLP, Southern blot, TaqMan [7,15,16], microarray [5], Multiplex ligation-dependent probe amplification (MLPA) [17], digital droplet PCR (ddPCR), pyrosequencing and paralogue ratio test [4,18,19,20]. Some of the available methods require large quantities of DNA, are time consuming [4] and have a higher degree of difficulty in data analysis and are prone to cross-contamination. 

A new strategy, named Gene Ratio Assay Copy Enumeration (GRACE)-PCR was developed and validated for α-globin gene rearrangement detection. It is a simple, closed-tube assay that allows direct visual identification of copy number variations [21]. Therefore, we developed a widely accessible method based on this HRM-GRACE-PCR assay to determine *C4A*, *C4B*, *C4S*, and *C4L* gene copy number polymorphisms.

## 2. Results

### 2.1. C4 CNV GRACE-PCR Design and Optimization

Initially, primers at different concentrations were assayed. For the C4A/ RP1, C4B/ RP1 and C4L/ RP1 primer pairs, amplification of the reference gene RP1 was very efficient; thus, it was necessary to increase the concentration for the C4A, C4B and C4L primers. In contrast, for the C4S gene, it was necessary to double RP1 primer concentration in relation to C4S primers. DNA samples of known C4 DNA genotype (Table 1) were used to validate the copy number and determine the melting temperature of each amplicon. 

AS haplotype carrying only the *C4A* short isoform. BS haplotype carrying only the *C4B* short isoform. AL haplotype carrying only the long *C4A* gene. BL haplotype carrying only the long *C4B* gene. The order is not determined and they are listed alphabetically. 

All five pairs of primers successfully amplified the corresponding genes with dissociation of a single peak. The negative control produced no dissociation peak. The HRM assay resulted in two distinguishable peaks with melting temperatures described in Table 2. Peak melting temperatures ranged between ± 0.1 °C. 

**Figure 2:**
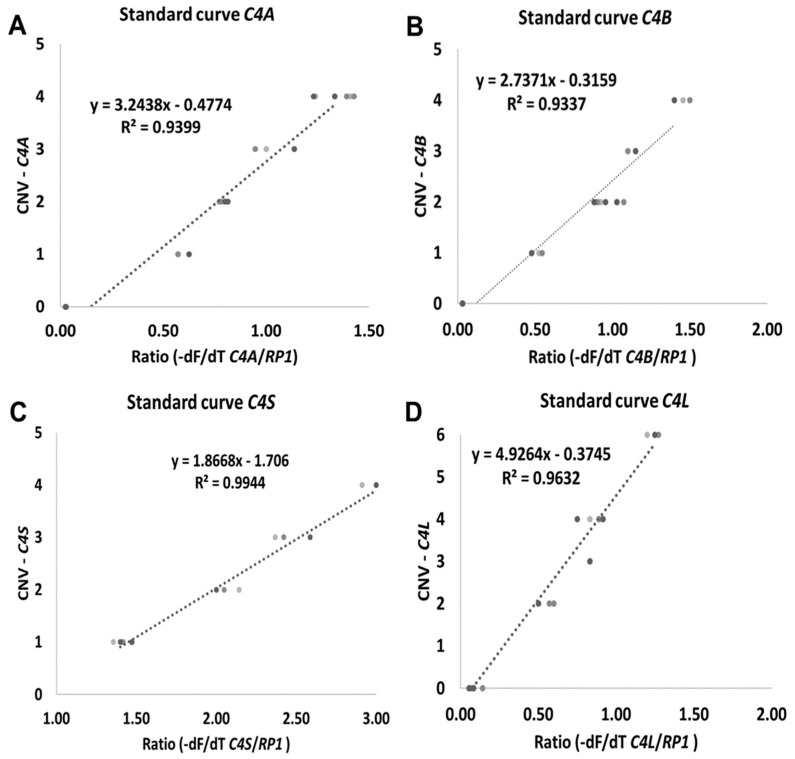
Standard curves generated by plotting ratio ((-dF/dT C4/RP1 genes) against CNV. (**A**) *C4A* gene. (**B**) *C4B* gene. (**C**) *C4S* gene and (**D**) *C4L* gene. -dF/dT (negative first derivative of the normalized fluorescence/first derivative of the temperature).

Different target DNA concentrations were assayed from 5 to 25 ng of genomic DNA. The target DNA concentration was optimized to ensure maximal specificity. The best outcome was observed at 10 ng/µL in comparison with higher concentrations (data not shown). Linear functions were calculated by plotting the -dF/dT *C4*/*RP1* ratio against known target C4 gene copy numbers. For all reactions correlation coefficients were always greater than 0.90 (Figure 2). C4 copy number from heathy blood donors was calculated from the applied equation for each isoform (Figure 2A–D).

### 2.2. Validation of the C4 CNV Determined by HRM-PCR/GRACE-PCR Assay

**Figure 3:**
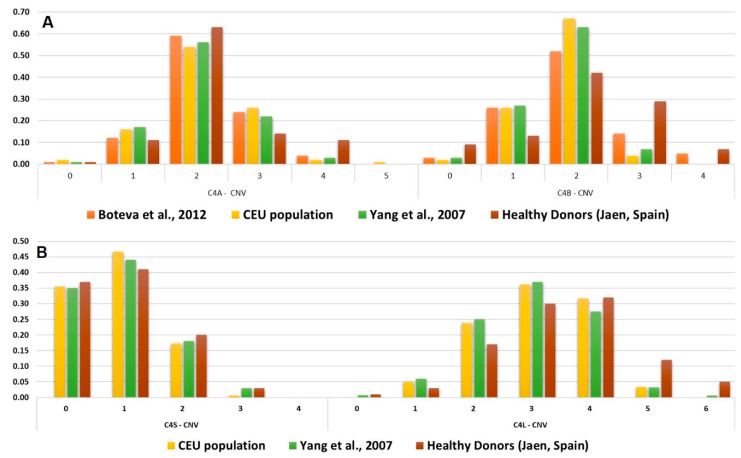
Reported *C4* genes copy number frequencies. (**A**) Frequency distribution of *C4A* and *C4B* CNV in Spanish populations and healthy controls. (**B**) Frequency distribution of *C4S* and *C4L* CNV in Spanish populations and healthy controls. CEU population corresponds to Utah residents (CEPH) with Northern and Western European ancestry.

The assay was validated with 72 DNA samples from anonymous healthy blood donors. Each target *C4* gene (*C4A, C4B, C4S, C4L*) was genotyped in a 48-well plate, including 19 unknown samples, four control samples with known C4 haplotypes and a negative control, all the samples were tested in duplicate. The range of C4 copy numbers in Spanish blood donors was similar to the distributions previously described in cohorts of individuals of European ancestry [18]. The distribution of C4A, C4B, C4S and C4L copy numbers are shown in Figure 3, compared to previously genotyped populations.

## 3. Discussion

We describe a widely accessible procedure to study *C4* copy number variation polymorphism, where the overall time of analysis is approximately 2 hours. The system consists of a combination of HRM with GRACE-PCR methods, which allow for detection and quantification of *C4* gene copy numbers for the *C4A*, *C4B*, *C4S* and *C4L* isoforms, using the peak ratio between the *C4* genes and a reference gene that correlates with the variation in the number of copies. A concurrent analysis of the standard curve with samples of known genotype increases result reliability with exact *C4* gene copy number quantification. Additionally, because the change in peak heights was not linear, intra-assay standards were necessary [22]. Analyzed healthy donors (*n* = 76) displayed a normal *C4* CNV distribution pattern ranging from 1–6 total copies, as has been previously described in other studies. A healthy control with complete deficiency of *C4A* and *C4B* components was found, which is very rare in this population. Previous studies have demonstrated that complete or partial deficiencies of *C4B* are associated with susceptibility to infectious diseases [23]. Additionally, it has been reported that *C4A* and *C4B* copy number variations are related to autoimmune disorders. This has led to the development and validation of a wide variety of assays to quantify the copy numbers of these genes and establish disease associations with *C4* CNV in genetic epidemiological studies.

Turner et al. (2015), [21] developed a methodology named the GRACE-PCR, a screening test for detection of deletions and duplications of the α-globin genes. This is a closed-tube technique with lower contamination risk, faster, cheaper and simpler than other assays. Following this, Turner et al. (2016) [24] used HRM-PCR/GRACE-PCR assay to detect all common point mutations and larger rearrangements of the hemoglobin subunit beta (*HBB*) gene. They detected 44 distinct pathological genotypes, resulting in a primary, quick, sensitive, specific and cost-effective screening test. The system herein described for *C4* CNV diagnostics, broadens HRM applications for gene dose quantification, increasing the range of laboratories that can perform *C4* gene dosage. 

According to Szilagyi et al. (2006) [25] quantification of *C4A* and *C4B* genes is clinically relevant, because there is a strong association between copy number variations and autoimmune disease susceptibility.

## 4. Materials and Methods

### 4.1. Samples

Eight genomic DNA samples of known *C4* genotype were used. Samples were obtained from subjects who deposited their DNA in the International Histocompatibility Working Group DNA Bank (IHWG) (Seattle, Washington, USA). Samples were selected to represent all *C4* possible copy number variations (CNV) from 0 to 6 copies and different haplotypes (Table 1). The assay was validated with 72 randomly selected samples of unknown genotype from anonymous healthy blood donors from Blood Transfusion, Tissues and Cells Bank of Transfusion Blood Center Jaén (Jaén, Spain). In total 80 samples were analyzed. Using a Taqman qPCR assay DNA samples of a known genotype were previously tested for copy number [15]. The study was designed and performed according to the Declaration of Helsinki and was approved by the Institutional Review Board of the Universidad de Jaén and Hospital Ciudad de Jaén (code: MAY.16/2, 30 May 2016). All donors provided written informed consent.

### 4.2. Blood DNA Extraction

Blood samples were collected from healthy donors. Genomic DNA was extracted as previously described and diluted to 100 ng/µL and stored at -20°C until use [26]. 

### 4.3. DNA Quantification

DNA quantity and quality was determined spectrophotometrically. For the PCR, DNA concentration was adjusted to 10 ng/µL., with an A_260_/A_280_ purity > 1.7. 

### 4.4. qPCR Oligonucleotides

The single-copy gene serine/threonine kinase 19 (*STK19* also known as HLA-*RP1*, here referred to as *RP1*) was selected as an endogenous reference gene (located at 6p21.33). In a diploid genome it is always present in two copies, acting as normalizing gene. The *RP1* gene has no known sequence polymorphism or duplicated regions in the human genome [15].

Primers for *C4A*, *C4B*, *C4S* and *RP1* genes were previously described [15]. Forward primer used for both the *C4S* and *C4L* assays was used as reference. *C4L* gene Reverse Primer was designed using Primer3Plus (Boston, MA, USA) (http://www.bioinformatics.nl/cgi-bin/primer3plus/ primer3plus.cgi). Primers were selected to generate products with different melting temperatures, ranging approximately 3 to 5 °C, with similar annealing temperatures and minimal homology with each other. All primers were verified for specificity using NCBI (Bethesda, MD, USA) Primer BLAST program (http://www.ncbi.nlm.nih.gov/ tools/primer-blast). High performance liquid chromatograph (HPLC) purified primers were commercially synthesized (Metabion international AG, Planegg, Germany). Primer sequences are presented in Table 2.

### 4.5. Number of PCR Cycles

To limit amplification saturation during the PCR’s exponential phase, reactions of 24, 26, 28 and 30 cycles were performed. Thus, ensuring the amount of each PCR product matched the one present in the analyzed sample to avoid an increase in initial copy number [21].

### 4.6. Optimization of HRM-PCR/GRACE-PCR Assay

PCR was performed using an Illumina’s Eco Real-Time PCR System (San Diego, CA, USA). Each plate contained four control samples for result validation and a standard calibration curve. All samples were tested in duplicate on at least three different days. The run assay had four HRM- GRACE-PCRs each with two primer pairs, C4A-*RP1*; C4B-*RP1*; C4S-*RP1* and C4L-*RP1*. All reactions were performed in a final volume of 12 µL, containing 10 ng of genomic DNA, 6 µL of Precision Melt Supermix (Bio-Rad, Hercules, CA, USA), and the following primer pairs (Table 3). Final volume was adjusted to 12 µL with molecular grade water. Negative controls (PCR-grade water) were included in each PCR reaction to verify for contamination.

### 4.7. Standard Curve

**Figure 4:**
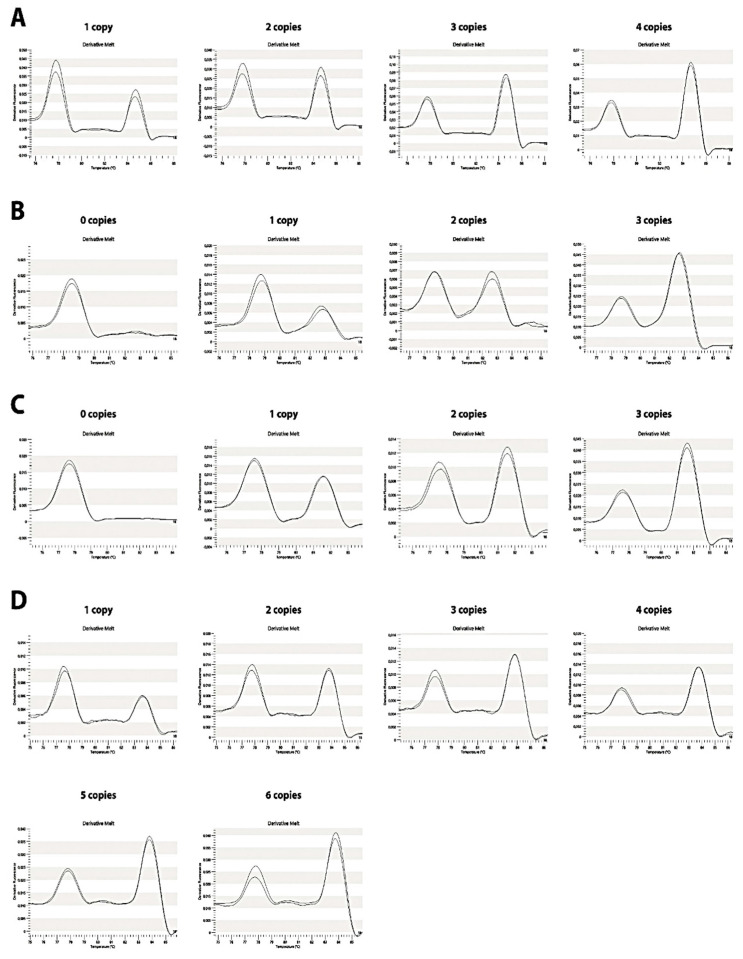
Pattern for *C4* genes copy number. (**A**) *C4A*, (**B**) *C4B*, (**C**) *C4S* and (**D**) *C4L* genes. Peak height (-dF/dT) versus temperature plots, relative to the reference gene. The melting plots are proportional to the copy number of each gene.

To calculate the CNV of genomic DNA samples, a standard curve for each of the target genes was performed. Curves were generated by plotting *C4* vs. *RP1* peak ratio values against CNV for each gene (Figure 2 and Figure 4). To ensure the validity of the assay, control genomic DNA obtained from the International Histocompatibility Working Group (IHWG) with known *C4* CNV (from 0 to 3 in *C4A*, *C4B* and *C4S* and from 0 to 5 in *C4L*) was used as standard (Figure 2).

The ratio was calculated by dividing *C4* gene and *RP1* maximum peak height value. This resulted in a plot in which the peak heights between *RP1* and *C4* melting regions were dependent on C4: *RP1* gene copy number ratio (Figure 4). For unknown genotype samples the same normalization procedure was performed to obtain ratio and copy number.

### 4.8. Data Analysis

Data acquisition and analysis were performed using Illumina’s Eco Real-Time PCR System software EcoStudy version 5.0 and Microsoft Office Excel. The amount of PCR amplification from *C4* and *RP1* genes is proportional to the height of the peak on the -dF/dT versus temperature plots. In addition, an internal control was conducted for each sample from an individual, where the sum of *C4A* copies plus the *C4B* copies should equal the sum of *C4L* copies plus *C4S* copies. The intra-assay coefficient of variation was determined by triplicate testing of the control and donor samples. 

## 5. Conclusions

In conclusion, the assay developed in this study offers an attractive alternative for rapid, sensitive and specific quantification of *C4* genes’ CNVs. 

## Figures and Tables

**Table 1:** Selection of human cell lines with known C4 CNV genotype [6,15].

Cell Line.	*C4A*	*C4B*	*C4S*	*C4L*	Haplotypes
COX	0	2	2	0	BS BS
WT51	4	0	0	4	AL-AL AL–AL
MADURA	2	2	4	0	AS-AB AS-BS
CB6B	4	4	2	6	AL-AL-BL-BS AL-AL-BL-BS
WT8	1	2	0	3	AL-BL BL
DAUDI	2	1	1	2	AL-BS AL
MANIKA	2	3	3	2	AL-BS-BS AL-BS
HOM2	3	2	1	4	AL-AL-BS AL-BL

**Table 2:** *C4* gene primers designed and selected for the HRM-PCR/GRACE-PCR assay. Amplicon size and melting temperature.

Target	Primers	Primer Sequence (5’ →3’)	Amplicon Size (bp)	Amplicon Tm (°C)
*C4A*	*C4A*-F	CCTTTGTGTTGAAGGTCCTGAGTT*	141	84.8
	*C4A*-R	TCCTGTCTAACACTGGACAGGGGT*		
*C4B*	*C4B*-F	TGCAGGAGACATCTAACTGGCTTCT*	86	81.6
	*C4B*-R	CATGCTCCTATGTATCACTGGAGAGA*		
*C4S*	*C4S*-F	TTGCTCGTTCTGCTCATTCCTT*	103	81.5
	*C4S*-R	GGCGCAGGCTGCTGTATT*		
*C4L*	*C4L*-F	TTGCTCGTTCTGCTCATTCCTT*	133	83.7
	*C4L*-R	CCAATGGACTTCAGGAACCC		
*RP1*	*RP1*-F	GACCAAATGACACAGACCTTTGG*	79	77.6
	*RP1*-R	GACTTTGGTTGGTTCCACAAGTC*		

Note: * [15].

**Table 3:** Primer pairs for high resolution melting (HRM)-PCR/gene ratio analysis copy enumeration (GRACE)-PCR assay. Gene, final concentration of each primer required for HRM-PCR/GRACE-PCR.

Gene	Final Concentration	Tube
*C4A*	0.5 µM	Tube 1
*RP1*	0.33 µM	
*C4B*	0.21 µM	Tube 2
*RP1*	0.16 µM	
*C4S*	0.25 µM	Tube 3
*RP1*	0.5 µM	
*C4L*	0.5 µM	Tube 4
*RP1*	0.25 µM	

## Abbreviations

*C4A*	Complement component *C4A*
*C4B*	Complement component *C4B*
*C4L*	long *C4* gene
*C4S*	short *C4* gene
CNV	Copy Number Variation
GRACE	Gene Ratio Analysis Copy Enumeration
HERV-K	Polymorphic endogenous retrovirus insertions
HRM	High Resolution Melting
*STK19*	serine/threonine kinase 19 (*STK19*), also known as HLA-*RP1*, here referred to as *RP1*
